# Functional Markers for Precision Plant Breeding

**DOI:** 10.3390/ijms21134792

**Published:** 2020-07-06

**Authors:** Romesh K. Salgotra, C. Neal Stewart

**Affiliations:** 1 School of Biotechnology, Sher-e-Kashmir University of Agricultural Sciences & Technology of Jammu, Chatha, Jammu 190008, India; 2 Department of Plant Sciences, University of Tennessee, Knoxville, TN 37996, USA

**Keywords:** functional markers, functional sequence characterization, precision plant breeding, elite cultivar

## Abstract

Advances in molecular biology including genomics, high-throughput sequencing, and genome editing enable increasingly faster and more precise cultivar development. Identifying genes and functional markers (FMs) that are highly associated with plant phenotypic variation is a grand challenge. Functional genomics approaches such as transcriptomics, targeting induced local lesions in genomes (TILLING), homologous recombinant (HR), association mapping, and allele mining are all strategies to identify FMs for breeding goals, such as agronomic traits and biotic and abiotic stress resistance. The advantage of FMs over other markers used in plant breeding is the close genomic association of an FM with a phenotype. Thereby, FMs may facilitate the direct selection of genes associated with phenotypic traits, which serves to increase selection efficiencies to develop varieties. Herein, we review the latest methods in FM development and how FMs are being used in precision breeding for agronomic and quality traits as well as in breeding for biotic and abiotic stress resistance using marker assisted selection (MAS) methods. In summary, this article describes the use of FMs in breeding for development of elite crop cultivars to enhance global food security goals.

## 1. Introduction

At the core of traditional plant breeding is phenotypic selection of superior genotypes. In reality, selections are the product of genetic recombination (genotype) and environment interactions. In plant breeding, genetic variability is the base for the improvement and the development of new cultivars. Genetic variability is repetitively produced in crosses and selections for desired traits. As we select beyond morphological traits to include genetic markers such as cytological and biochemical markers, more than 12 years may be needed to develop a variety. Herein, cataloging new genes and allele variants was initially accomplished using these markers and tracked in subsequent crosses. These days, these traditional markers have largely been supplanted by those from genomics approaches [1,2,3]. Genomic markers are not affected by environmental factors, sample collection stages, and the developmental stages of the plant [4].

Under a 50% higher demand for food by 2030 [5] from limited genetic and environmental resources, plant breeders are challenged to increase their output of superior varieties that are adapted to changing climates [6]. Further, the genetic base has narrowed with the introduction of high yielding varieties with a commensurate yield plateau [7]. Under these circumstances, plant breeders must explore and implement a wide range of genomic resources. There is a plethora of new breeding technologies and approaches that address global food security challenges, which simultaneously include both sustainability and humanitarian goals [8].

Precision breeding is a plant breeding approach in which a phenotypic trait of interest is selected by means of identifying a functional marker (FM) that is directly derived from the genomic region of a trait-controlling gene [9]. In precision breeding, selections are based on the polymorphic genic regions linked with a trait of interest. Availability of genomic resources are of utmost important for development of FMs and their use in precision breeding [10]. At the core of genomic innovations are complete genome sequences of cultivated crop species. In addition, systems biology approaches are used to infer relationships among transcripts, genes, proteins, and phenotypes [11]. Hence, hosts of molecular markers have been developed and are being used in plant breeding programs for genotype identification, phylogenetic studies, population structure and diversity analysis, and to better understand genome evolution [12,13].

## 2. Brief History of Molecular Marker Development

The genomics revolution during the 1990s greatly improved our understanding of the genetic make-up of a wide array of living organisms, including plants. With the advent of molecular marker technology, plant breeding became more efficient, including identifying quantitative trait loci (QTL) for use in genetic mapping [13]. Different types of molecular markers have been developed: restriction fragment length polymorphism (RFLP) [14], random amplification of polymorphic DNA (RAPD) [15,16], amplified fragment length polymorphism (AFLP) [17], microsatellite or simple sequence repeat (SSR) [18,19], sequence characterized amplified region (SCARs) [20], cleaved amplified polymorphic sequences (CAPS) [21], single nucleotide polymorphism (SNP) [22], and diversity arrays technology (DArT) markers [23]. Among these, SNP markers have gained the widest use [24]. Most of these marker types are random DNA markers (RDMs), which may be lost during recombination. In contrast, if candidate trait genes are known, then DNA markers (functional markers; FMs) within the gene or closely linked to the gene can be developed and used in agricultural crops. Herein lies the power of FMs.

**Figure 1:**
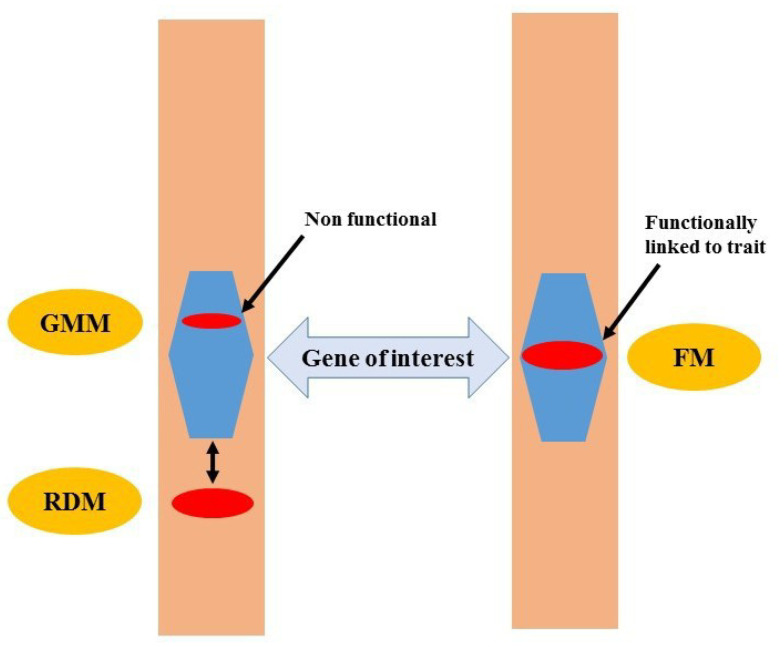
Functional markers (FMs) are functionally linked with phenotypic traits compared to random DNA markers (RDMs) and genic molecular markers (GMMs).

FMs are defined as DNA markers that have been derived from functionally characterized sequence motifs [25]. Therefore, SNPs as FMs are more useful in plant breeding compared with RDMs and genic molecular markers (GMMs) (Figure 1). Although GMMs may be present within a gene of interest, functionally, they may not be linked to the phenotypic trait of interest, which may lead to false selection in MAS. FMs have been called “perfect markers” in contrast with RDMs as “non-perfect markers” such as RFLPs, AFLPs, or SSR markers [26]. Thus, this review assesses the state-of-the-art of FMs and their use in plant breeding.

## 3. Functional Markers

FMs in genic regions are optimally directly linked with a phenotypic trait, i.e., one that functionally characterizes the observed phenotypic variation [25]. FMs are also known as precision markers or diagnostic markers. FMs enable efficient and quick characterization and screening of germplasm for allelic diversity with accuracy since they are not subjected to recombination. Once genetic effects have been assigned to functional sequence motifs, FMs can be used for fixation of beneficial alleles in the breeding population [26]. As the polymorphism occurs within the target gene and fixes the favorable allele in the breeding population, the utility of FMs can also be made in selection of complex traits [27].

**Figure 2:**
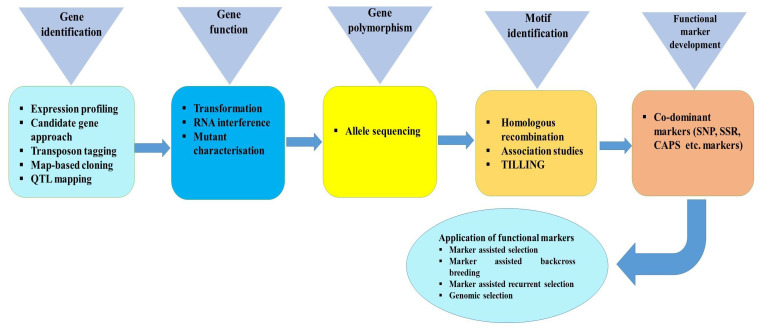
Different approaches involved in development of functional markers (FMs).

The development of FMs first requires the identification of a gene of interest affecting the phenotypic trait, its nucleotide sequence, and functional characterization [13]. Gene identification may be attained by a number of approaches such as expression profiling, map-based cloning, QTL mapping, expression profiling, and transposon tagging [28,29] (Figure 2). Plant transformation for overexpression or knockdown analysis is necessary for functional characterization of a candidate gene [30]. The second step towards FM development involves the study of allelic variation within characterized genes. Then, allele sequencing must be performed between genotypes to identify those polymorphisms that underlie causative variation of the phenotypic trait [31]. The development of FMs requires critical understanding about the sequence of allele/gene linked to the trait. These polymorphisms among alleles for genes of interest may be due to insertions or deletions (InDels), SNPs, different number of repeat motifs within SSRs, and partial or complete loss of the gene [28]. Hence, FMs may be based on a wide range of types of coding and non-coding DNA [32].

Over the last two decades with advancement of different next generation sequencing (NGS) platforms, numerous QTLs have been identified by developing various mapping populations such as recombinant inbred lines (RILs), near isogenic lines (NILs), doubled haploids (DHs), etc. Development of mapping population by crossing two diverse parents is time and cost consuming. To overcome these barriers, an alternate approach, association mapping, has been used for detecting QTL in natural or breeding germplasm. Association mapping (AM) is a powerful approach that identifies polymorphisms near or within a gene of interest that controls the phenotypic differences between genotypes [33]. Linkage disequilibrium (LD) tends to be maintained over many generations between loci that are genetically linked to one another, which enables marker assisted selection (MAS) [34]. Association mapping also facilitates the search for functional variation in genes of interest; the larger and more diverse the samples are, the greater the potential associations are [35]. Genic SNPs and maintenance of LD are quite helpful for FM development and subsequent marker-assisted backcross breeding (MABB) activities [36].

High-throughput genotyping technologies and large-scale genomic resources have led to ample SNP candidates for high-resolution linkage map production [37]. Furthermore, genome-wide association studies (GWAS) are now a viable alternative to QTL mapping for dissecting important quantitative traits. GWAS may provide efficient assessment representative sets of individuals and genes. GWAS leverage LD to enable high density genotyping that spans the entire genome of an organism to identify genomic regions linked to the phenotypic traits. Recently, GWAS have been successfully applied for identification of genomic regions associated with important traits in rice, barley, corn, wheat, and other crop species. GWAS in these crops have provided important information about the marker-trait associations, which can be successfully used in plant breeding programs [37]. Recently, GWAS have also been used in an association mapping population for identification of SNPs associated with total sugar content and sorbitol for improving fruit quality of lettuce [36] and peaches [38].

Advances in next-generation sequencing technologies have driven the costs of DNA sequencing down to the point that genotyping-by-sequencing (GBS) is now feasible for plant species with both high genetic diversity and large genomes [39] to the degree that they have become breeding tools. GBS involves the use of restriction enzymes for targeted complexity reduction of the genome followed by multiplexing and sequencing. GBS can generate numerous SNP markers covering much of the genome in a cost-effective manner [39,40]. Therefore, these genome-wide SNPs can be used in genomic selection, GWAS, and genetic diversity studies. In one example, GBS-derived SNPs associated with the functional allele *E3Ha* for maturity in soybean has been identified [41].

Similarly, transcriptome sequencing provides a new tool for genomic studies on model or non-model organisms. RNA-sequencing (RNA-Seq) is a powerful tool for transcription profiling, providing rapid access to a collection of expressed sequences. Transcriptome sequencing of an organism provides facile insights into the gene space, enabling gene discovery and FM development as well as study gene expression patterns and comparative biology [42]. RNA-Seq may enable molecular marker development, including FMs in non-model plant species that may not have a reference genome yet sequenced [43]. RNA-Seq has been successfully applied in different domains of life from yeast to plants. SNP detection is an important part of molecular genetic research because SNP loci can be exploited to construct high-density genetic maps and enable GWAS [44,45,46,47,48,49].

**Figure 3:**
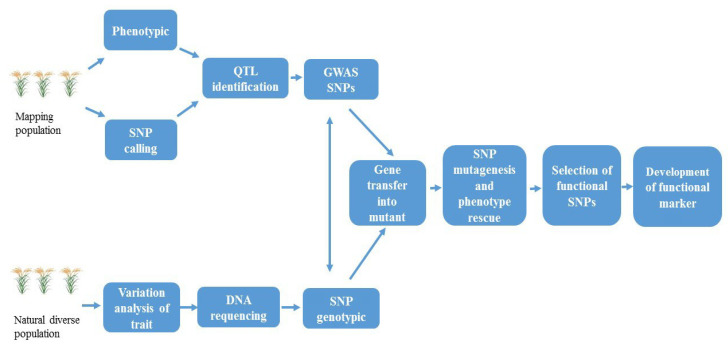
Genome-wide association studies (GWAS) for identification of functional single nucleotide polymorphisms (SNPs) and development of FMs.

Functional SNPs are useful for improving breeding efficiency. With the progress in functional genomics research, increasing numbers of FMs responsible for agriculturally important traits have been identified, which provide valuable genetic resources for molecular breeding. Resequencing and SNP genotyping are two key strategies used in GWAS for identification of functional SNPs and development of FMs (Figure 3). Mapping populations (RILs, DHs, segregation, etc.) are used for appropriate QTL identification, in which genome resequencing of different lines generates saturated SNPs. The SNPs located at the QTL are regarded as GWAS SNPs, because the candidate genes at the QTL locus are predicted according to GWAS analysis. On the other hand, in association mapping, diverse germplasm is useful, and SNP genotyping on the basis of genomic resequencing provides a strong tool for the detection of SNPs in large accession collections. The comparison of GWAS SNPs from populations helps identify functional SNPs linked to the phenotypic trait.

Functional genomic techniques such as RNAi, site-directed mutagenesis, gene knockout analysis, and transposon tagging are useful in gene discovery [50]. With the possible exception of naturally occurring transposon systems in maize, most methods (transposon, T-DNA, antisense, and RNAi) rely on transgenic introduction of foreign DNA, which is not possible in most of the important crops. The development of transgenic is the main impediment in the use of functional genomics in the development of FMs in crops. However, RNAi has been exploited in the functional analysis of the 22-kD maize zein storage protein [51] and lysine rich in maize [52], the functional role of *GhACT1* gene in fiber elongation in cotton [53]. However, RNAi generates unpredictable outcomes, and the whole procedure is laborious, as it requires vector construction, transformation, and transgenic analysis [54]. RNAi has several limitations such as partial and short-term suppression of genes rather than rendering a complete loss-of-function. In addition, off-target effects may cause false positive observations [55]. Still, RNAi experiments with a range of target gene suppression, which enables knowledge about the effect of gene expression in target tissues, i.e., gene function [56].

The emergence of different genome editing tools such as clustered regularly interspaced short palindromic repeat (CRISPR-Cas9), zinc finger nucleases (ZFNs), and transcription activator-like effector nucleases (TALENs) is important, as these are breakthrough technologies to knock-out genes. CRISPR-Cas9 approaches are especially relatively facile, affordable, and efficient research tools [57]. CRISPR-Cas9 produces double stranded breaks (DSBs) to the target loci, which can be repaired via homology directed repair (HDR) or non-homologous end joining (NHEJ) mechanisms. In most of the cases, NHEJ causes deletion mutations or random insertion of variable lengths, resulting in knockout mutants with frameshift mutations. Most often, coding regions of genes are disrupted, leading to a loss of an endogenous protein. This tool has numerous advantageous compared with RNAi technology, such as complete loss of function with relatively low off target activities [56], heritable and permanent or stable knock outs, and efficient characterization of non-coding RNAs (ncRNAs) by disrupting their DNA coding sequences. In addition, CRISPR-mediated knockouts in various crops [56,58] are likened to natural mutants and can be used directly in breeding and rapid crop domestication.

Point mutations for knock-in mutations may also result in gene gain-of-function; thus, using CRISPR-Cas9 in this way is also a breeding tool. HDR-mediated knock-ins have been achieved in rice, cotton, tobacco, poplar, sweet potato, and several other plant species [59]. 

Compared to different functional genomic approaches, targeting induced local lesions in genomes (TILLING) is a non-genetically modified organism (GMO) techniques and applied to any plant species regardless of genome size, ploidy level and mode of propagation. TILLING offers many advantages in cases where the transformation is difficult or if the investigation of a continuing series of unknown genes in a specific crop is desired. This technique requires no complicated manipulations or expensive apparatus. It enables one to screen the mutant pools easily for investigating the functions of specific genes, avoiding both confounding gene separation steps and tedious tissue culture procedures as are involved in anti-sense RNA and RNAi. It allows rapid and inexpensive detection of induced point mutations in populations of mutagenized individuals. Moreover, TILLING involves a series of alleles in a targeted locus compared to functional genomic approaches. In TILLING, the use of ethyl methanesulfonate (EMS) chemical mutagen produces G/C to A/T transition, which provides high frequency of point mutations distributed randomly in the genomes. Endonuclease cut effectively with the multiple mismatches in a DNA duplex and the heteroduplex DNA of unknown sequence to that of a known sequence reveals the positions of polymorphic sites. Therefore, both nucleotide changes and small insertions/deletions are identified. It can be performed with fewer expenses than the full sequencing methods currently used for most SNP discovery. Moreover, a well developed and tested protocol of TILLING is available for a number of crops, such as lotus [60], barley, common bean, field mustard, maize [61], oat, pea, peanut, potato, rice [62], rape seed, sorghum, soybean, *Medicago* spp., tomato, and wheat [63]. TILLING is an attractive strategy for a wide range of applications from the basic functional genomic study to practical crop breeding.

Regardless of the source of DNA, once FMs are developed, the next step is the functional validation of markers regarding the link to a gene of interest [64]. The validation of newly developed FMs for their functionality can be carried out by gene expression studies, including virus induced gene silencing (VIGS) and gene knock-down and knock-out analyses as noted above [65,66,67]. However, the VIGS technique has an advantage over the other techniques when it is useful to silence multiple genes within gene families, which has additional power in the analysis of polyploid species [65,68,69].

## 4. Advantages of FMs over Other Markers

RDMs are the most prevalent DNA markers used for indirect selection. RDMs are derived from the DNA sequence polymorphisms in the regions adjacent to the gene of interest; they do not always lead to predictive selection for traits of interest. However, the problems associated with the use of RDMs can now be overcome by the use of FMs that are 100% predictive of the corresponding phenotype. Once genetic effects have been assigned to functional sequence motifs, FMs fix alleles in several genetic backgrounds without additional calibration [70,71]. This is advantageous in marker applications, particularly in plant breeding, to select parental materials to build segregating populations as well as subsequent selection of advanced breeding lines [25].

FMs reside within the target genes themselves and are directly linked to the morphological traits that can be used with great reliability and efficiency to identify favorable alleles in a breeding program [36]. FMs also reduce the chances of loss and false selection of information using MAS [65,72]. Additionally, in MAS, QTL validation is needed when applied to different genetic backgrounds; however, FMs avoid this validation [30,65,73]. FMs can facilitate the selection of exceptional phenotypic traits that would enable a breeder to identify rare recombinants in a large population [74]. In addition, FMs are useful for screening for alleles in natural as well as breeding populations, fixation of alleles in the population, construction of linked FM haplotypes, and combination of FM alleles associated with complex traits [25].

## 5. FMs in Precision Plant Breeding

Genome sequencing costs continue to decrease, which has facilitated FM development. Since FMs outperform RDMs, FMs are increasingly being used in MABB activities for quantitative and quality traits [74,75]. In precision breeding, FMs can be used in germplasm evaluation, genetic diversity analysis, MAS, MABB, marker assisted recurrent selection (MARS), and genomic selection (GS) for improvement of important traits [29,76,77,78].

### 5.1. Germplasm Evaluation and Genetic Diversity

Plant genetic resources (PGR) are the basic material required for the improvement of crop species. These are the important sources of gene(s) for yield enhancement, quality improvement, disease and insect pests, as well as abiotic stresses. Before the advent of plant genomic approaches, the main genetic markers for evaluation of germplasm were various morphological traits [17]. Sequencing revolution enables the development of numerous molecular markers such as RDMs, GMMs, and FMs [79]. To screen the germplasm for allelic variation for particular phenotypic traits, FMs enable direct linkage with the gene of interest and can be used directly in breeding programs [80]. FMs enable the characterization and screening of the germplasm for allelic diversity with more accuracy. Compared to RDMs, FMs identify agronomically important genes directly from germplasm such as landraces, traditional cultivars, wild relatives, and plant genetic resources. The identified traits in the germplasm are linked with particular FMs and can be utilized in crossing programs for development of new cultivars. This also enables the plant breeder to develop new genetic resources to act as bridging genotypes for transferring the valuable genes to cultivated ones [81]. Besides this, the International Union for the Protection of New Varieties of Plants (UPOV) also endorsed the use of FMs for trait-specific characterization of germplasm and varieties for distinctness, uniformity, and stability (DUS) characters [82].

The knowledge of genetic variability present within and among the germplasm is the basis for the improvement and the development of crop varieties [83]. Genetic diversity information of PGR is essential and prerequisite for breeding programs. Today, vast PGRs that are available are characterized based on the phenotypic traits, but few of these have been characterized at the molecular level. Availability of high throughput genotypic techniques made large-scale use of molecular markers in genetic diversity analysis, identification of variety, conformity of F_1_ hybrid, and in plant variety protection [84,85]. For identification of potential and diverse genotypes for breeding, a broad genetic base is required [2,86]. The genomics era has provided several genomic resources such as RDM, GMM, and FM to assess the genetic variability in the available genetic resources [87]. Trait-specific FM genetic diversity is essential to broaden the genetic base to enable precision breeding [88]. FMs hold the promise for identification of alleles/genes involving polymorphisms causing functional genetic variation [89]. FMs can be effectively used to characterize the genetic diversity among closely related plant species based on functionally characterized genes linked with the phenotypic traits [90]. Alternatively, genetic diversity among genotypes can be assessed in the functional parts of the genome by DNA-based profiling methods [91,92,93].

### 5.2. Marker Assisted Selection

**Figure 4:**
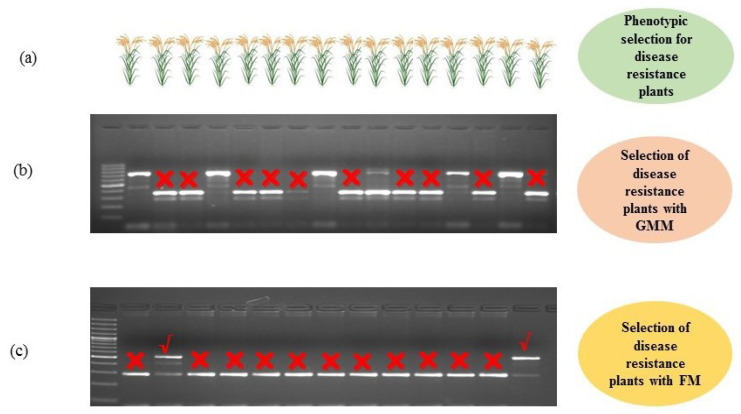
Use of functional markers (FMs) in marker assisted selection in precision breeding. (**a**) Phenotypic selection for disease resistance plants. (**b**) Selection of disease resistance using genic molecular markers (GMM). (**c**) Disease resistance plants selection using functional marker (FM).

MAS is a molecular breeding technique in which direct and indirect phenotypic selections of a genotype are made on the basis of a molecular marker that may consist of RDMs, GMMs, and/or FMs [2]. MAS is used in crop improvement to overcome the difficulties that arise from the conventional plant breeding methods [94,95]. FMs used in MAS help in selection and identification of genotypes in a segregating population, which are directly linked with morphological traits (Figure 4). FMs have the uniqueness for confirmation of candidate genes governing the desired phenotypic traits, which can be used directly in the plant breeding methods through MAS. Several FMs have been developed and used for improvement of important morphological, quality, and biotic and abiotic stress resistance traits in different crops such as wheat, maize, rice, fruits, and vegetables. In these crops, FMs have aided in the improvement of various qualitative and quantitative traits such as flowering time, photoperiod response, plant height, seed length, seed weight, aroma, amylose content, oil content, and resistance to various diseases and insect pests [64,96,97]. These traits may be controlled by recessive or dominant alleles of a gene of interest. 

MABB is used to transfer a phenotypic trait controlled by a recessive or dominant allele into an elite crop variety through repeated backcrosses and selections using molecular markers. In MABB, selection is operative both at genotypic as well as phenotypic levels. In MABB, the desired traits are transferred from the donor parent (non-recurrent parent) to a widely-adapted variety (recurrent parent). Repeated backcrossing and selection are practiced on the basis of molecular markers and the phenotypic traits until most of the genes stemming from the donor are eliminated. In MABB, FMs are used to target the gene controlling the desired trait of interest (foreground selection) and in recovering the genome of an elite variety (background selection) if a sufficient number of markers are available. FMs have been successfully employed to select for a number of genes controlling desired traits that have been transferred to adapted varieties via MABB [72]. The efficiency of MABB can be enhanced by using FMs to transfer the desired gene(s) controlling simple or complex trait(s) into cultivated varieties [98]. Moreover, FMs can be used for multiplex screening assays for foreground selection to identify introgressed genes [99,100].

MARS plant breeding methods for transferring complex traits into varieties take a long time under continuous recurrent selection. The phenotypic selection of complex traits may be difficult because of ambiguous phenotypic selection and analysis of complex traits owing to the large number of genes that contribute to the traits. In MARS, favorable alleles of complex traits are accumulated with the help of genetic markers and are thus made more efficient. Among the various genetic markers, FMs help the accumulation of favorable alleles of a complex trait most efficiently [101]. For improvement of complex traits, several recurrent selection cycles are required to accumulate favorable QTL alleles in the breeding population [102], which is aided by use of FMs. MARS thus decreases the number of needed breeding cycles while increasing the precision of selecting complex traits. When parents used in MARS are crossed when informed by FMs, an ideal genotype is possible after only a few successive generations of backcrossing. FMs enable genetic gain for the improvement of complex traits and the development of inbred lines of a hybrid [98].

### 5.3. Gene Pyramiding

**Figure 5:**
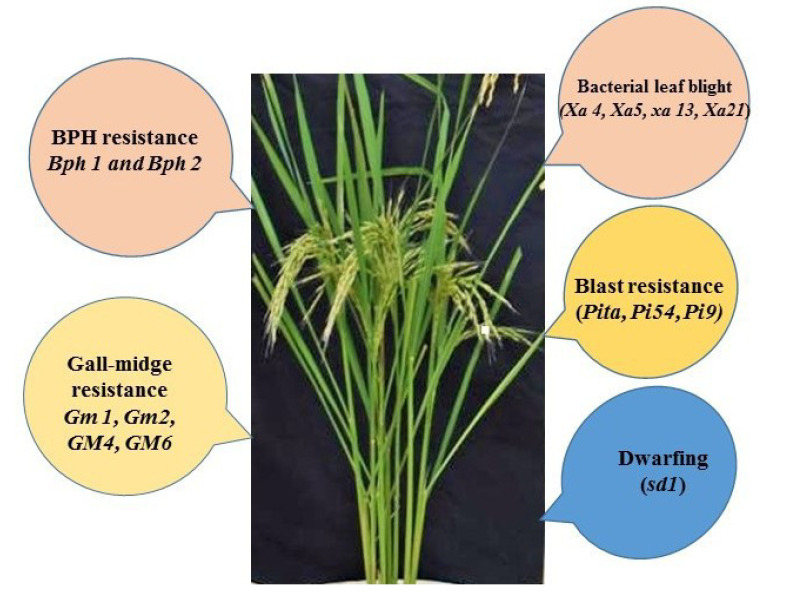
Pyramiding of desired genes in a cultivar using functional markers (FMs).

Gene pyramiding is a method of assemblage of different desirable genes from various donor parents into a single plant (Figure 5). In gene pyramiding, genes controlling different traits are simultaneously transferred into a single cultivar. Gene pyramiding is used for improving few unsatisfactory traits of a widely-grown elite variety, and these unsatisfactory genes are replaced with better genes. Although gene pyramiding is possible through conventional plant breeding methods, phenotypic selection and identification of a single plant containing more than one gene are very difficult. There are chances of loss of gene of interest from recombination and number of meiotic cycles, which may convolute plant breeding [103]. FMs can improve the prospects of gene pyramiding for different traits [104] as demonstrated by the plethora of FMs linked with a multitude of morphological traits, quality traits, and resistance to biotic and abiotic stresses for use in many crops (Table 1) [64,105].

### 5.4. Genomic Selection

Genomic selection (GS) was developed by Meuwissen et al. [106], which is an advanced method of MAS. GS has aided the improvement of complex traits such as grain yield and its components, quality traits, and abiotic stress resistance, which vary rapidly with generation of desired phenotypes by selection [107]. GS is also known as genomics-assisted breeding (GAB), which uses phenotypic data, genotypic data, and modeling to predict the genomic estimated breeding values (GEBVs) for each individual [108,109]. In this method, GEBVs are used to predict the genetic values of selected candidates predicted from high density of markers that depends on all the major and the minor molecular effects [110]. GS requires: (i) a diverse population used for development of a training population; (ii) genotypic as well as phenotyping analysis of the training population; (iii) genotypes with high values of GEBV to be selected on the basis of their genotypic data; (iv) a testing population composed of progeny of the genotypes used as study material that are taken as input for the GS model to yield GEBVs; (v) high GEBV values are selected again in genotypes; and iv) the selected genotypes are used as parents for continuous crossing and selection [111]. In GS, FMs have advantages over other markers as few numbers of trait-specific FMs are required for predicting GEBV values. GS has been used in several crops including wheat [112], maize [113], and *Brassica rapa* [114]. Increased prediction of GEBVs is facilitated by using FMs [115].

## 6. FMs for the Improvement of Agronomic Traits, Quality Traits, and Stress Resistance

Advances in sequencing techniques enable the identification of SNPs and indels linked with various economically important traits; FM development is thus enabled [64]. Indels may cause phenotypic variation from extensive genomic effects, which are accompanied by chances of elimination from natural selection [116]. Hence, SNP-derived FMs have advantages over indel-derived markers because the widely distributed nature of FMs throughout the genome [117,118]. FMs have been developed for various agronomic, quality traits, and biotic and abiotic stress resistances, which have been pyramided in different crops using MAS, MABB, MARS, and GS approaches [64] (Table 1).

### 6.1. FMs for Agronomic Traits

FMs perfectly discriminate alleles of a targeted gene, and FMs have been deployed for improvement of important agronomic traits through MABB. In wheat, more than 97 FMs have been developed and used in MAS [119]. Two FMs for *TaSUS2-2B* and *TaZds-D1* genes encoding grain weight have been developed, which can be used in MABB for wheat improvement [120]. Similarly, FM markers that are based on SNPs present in the eighth exon of the *TaGW2* gene have been developed for enhancing grain weight in wheat [121]. In wheat, the pre-sprouting of spikes results in low grain yield production and development of low quality products. An SNP-based CAPS marker was developed for *TaSDr* gene responsible for low spike sprouting in wheat crop under field conditions [122]. Two semi-dwarf genes, such as *Rht1* and *Rht2,* encode a protein involved in GA-signal transduction located on chromosome 4B and 4D of wheat and played a significant role in the Green Revolution. *Rht1* and *Rht2* are the result of point mutations to change the tall genotypes of wheat to semi-dwarf ones. FMs were developed for screening the semi-dwarf genotypes in wheat germplasm [123].

In rice, the erect panicle trait significantly contributes to increased yield for which an FM has been developed that is transferable to other rice varieties through molecular breeding [118]. Similarly, the semi-dwarf gene *sd1* on chromosome 1 in rice also played a role in the Green Revolution. This is the most widely deployed gene in modern rice breeding programs in the world for development of semi-dwarf varieties. The *sd1* gene FM was developed owing to a 280 bp deletion within the coding region of the *Os20ox2* gene that encodes the non-functional protein that leads to reduction in gibberellic acid (GA_3_) production in dwarf rice plants [124]. An FM enabled the transfer of *sd1* gene in Ranbir Basmati rice through MABB [125]. In the two-line system of hybrid rice production, photoperiod-thermo-sensitive genic male (PGMS and TGMS) sterility is essential. An FM has been developed for the *pms3 (p/tms12-1)* gene to transfer male sterility in other rice varieties using MABB [126].

In maize, an FM for reduced plant height along with early flowering has been developed for the *Dwarf 8* gene from a deletion of bases in the coding region of the gene. This marker has been used for screening of maize germplasm for early flowering time and reduced plant height [31]. In barley, an FM has been developed because of deletions in the intron region in *VRN-H 1* gene. This gene is responsible for regulation of spring growth habit and vernalization in barley [127]. In mustard crops, trichomes provide protection against number of insect pests and diseases. An FM for *BrpHL1,* a trichome-related gene, is used in mustard breeding [128]. Similarly, FMs have been developed for agronomically-important traits of legumes and vegetable crops (Table 1).

### 6.2. FM for Quality Traits

Quality traits are important for a variety of reasons, such as to meet consumer preferences. FMs have been successfully applied in MAS to improve the food quality of crops [72]. A wheat FM, YP7A, was developed for the *Psy1* gene, which is involved with yellow grain pigment [128]. Similarly, an FM has been developed for *TaZds-D1,* which is also responsible for increased yellow pigmentation in wheat [152]. Gluten proteins determine bread-making quality of wheat. An SNP-based FM for *Glu-B3* encoding low molecular weight gluten protein has been developed for improving the quality of wheat products [151].

In rice, particularly basmati, aroma is one of the most important components for determining the price in the world market. Basmati, the aromatic rice praised for its unique quality, is a connoisseur’s delight, which has a pleasant aroma and is nature’s gift. A set of FMs linked to a recessive *badh2* gene responsible for 1-acetyl-2-pyrroline (AP) production has been identified and exploited for screening rice germplasm for fragrance [154]. Grain length and grain breadth also determine the grain qualities of rice. Long grain length adds an aesthetic value in rice for appearance and fetches a higher price in the world market. For grain length in rice, an SNP-based FM has been developed for gene *GS3* encoding trans-membrane protein for use in rice breeding. The *GS3* gene governs grain elongation after cooking longitudinally, an important trait affecting the physical appearance of the rice grains [220]. In rice, intermediate amylose content is preferred by the consumers, and an FM has been developed for gene *Wx-in* encoding moderate amylose content for screening of rice germplasm [159]. Similarly, in sorghum, an InDel based FM has been developed for the *SbBADH2* gene responsible for fragrance. This FM has been used in identification of sorghum genotypes possessing high fragrance, which can be further used in sorghum breeding [165].

Maize is an important cereal crop of the world, which is used as both food and feed crop. The quality improvement in maize is important to enhance the nutritional values for humans and animals. To increase the nutritional values of maize, plant breeders are successfully exploiting the modern molecular techniques for screening the genotypes and their use in breeding. In this context, SNP-based FMs for *ZmcrtRB3,* which encodes a carotenoid hydroxylase, have been developed in maize [161]. This gene affects a-carotene content in maize and is successfully transferred to other maize varieties to enhance the pro-vitamin A content. Similarly, FMs have been developed for *crtRB1 and LcyE* encoding pro-vitamin A content in maize [163]. Sweetness is an important quality trait in maize and is encoded by the *sugary1* gene. For successful screening and transfer of this trait, an FM has been developed [162]. Maize oil is a desirable best vegetable oil because of its high smoking point while frying. A gene *DGAT1-2* has been identified for governing oleic acid quantity and oil content in maize. An FM has been developed for this candidate gene to screen maize germplasm [160].

In soybean, an FM for *Gmbadh2-1 and Gmbadh2-2* genes, which are responsible for fragrance production, has been developed [166]. 

Celery is an important vegetable crop, which is rich in minerals and vitamins such as A, C, K, potassium, folate, and flavonoids. In celery, high flavonoid content is encoded by *AgFNSI,* and an FM has been developed for screening germplasm for this trait to be used in breeding [168].

### 6.3. FMs for Biotic Stress Resistance

Crop yield losses are caused by various pests, including diseases and insects. Several disease resistance genes have been identified and transferred into elite cultivars with the help of FMs (Table 1). Wheat diseases include leaf rust, yellow rust, brown rust, and powdery mildew. FMs for disease resistance genes have been developed for pyramiding of different resistance genes into various wheat varieties [221]. In wheat, FMs for resistance to leaf rust, yellow rust, and powdery mildew, such as *Lr 34, Yr 18,* and *Pm 38*, have been developed and used for pyramiding into elite wheat varieties for durable resistance to these diseases [174,222]. Similarly, an FM has been developed for gene *Sr45* encoding wheat stem rust resistance to be used in MABB [175].

Rice crop yield may be decreased by various diseases and insect pests such as bacterial leaf blight, blast, brown spot, and brown hopper. With the accessibility of complete genome sequences of rice subspecies *indica* and *japonica,* a number of FMs, such as *Xa3, xa5, xa13, Xa21,* and *Xa38,* have been developed for bacterial leaf blight [103,176,177,178,179]. Similarly, FMs have been developed for blast resistance genes such as *Pit, Pi54 (Pik^h^), Pi35,* and *PigmR* [182,183,184,185]. These FMs have been used in MABB for foreground selection of various resistance genes for development of rice cultivars such as “Samba Mahsuri” [178,223], “Improved Pusa Basmati 1” [224], “Pusa 1121” [180], “Improved Tapaswini” [225], and “Ranbir Basmati” [125]. 

In tomato, an FM for *ACY,* a gene that confers tomato yellow leaf curl virus resistance was developed and used to produce leaf curl virus resistance tomato cultivars [190]. FMs for bacterial wilt and fusarium wilt have been developed in tomato to be used in breeding [191]. 

In watermelon, FMs have been developed for Zucchini Yellow Mosaic Virus (ZYMV) resistance gene *eIF4E* (197) and powdery mildew resistance gene *Pm* [198]. Similarly, an FM for controlling Cauliflower Mosaic Virus (CaMV) resistance has been developed for the *cmv6.1* gene in cucumber [200]. Oumouloud et al. [193] developed an FM for the *Fom 1* gene responsible for controlling fusarium disease in melon. An FM has been developed for *Rpf* gene encoding leaf scald resistance in sugarcane, which can be used for development of sugarcane varieties resistance to this disease [194].

### 6.4. FMs for Abiotic Stress Tolerance

Abiotic stress is one of the major unpredictable and uncontrolled factors in crop production. Abiotic stress resistance is a complex trait controlled by polygenes and depends upon the time and the severity of the stress components. All these unprecedentedly and uncontrolled factors of abiotic stress make it difficult to characterize and develop FMs for abiotic stresses. Very few FMs have been developed for abiotic stresses compared to biotic stresses; however, one goal is to discover functional variations linked to the abiotic stress traits (Table 1). In wheat, an SNP linked with the dehydration tolerance has been identified in the *TaMYB2* transcription factor gene; an allele-specific FM has been developed for use in MABB [208]. Similarly, two SNPs were identified in the *DREB1* gene in wheat, and an allele-specific FM has been developed [210]. Pandey et al. [209] developed SNP-based FMs for the *TaAQP* gene encoding drought tolerance, which was validated in wheat varieties. Similarly, an FM for the *TtASR1* gene encoding salt tolerance in wheat has been developed, which should be helpful to screen wheat germplasm for salt tolerance [211].

In rice, the phosphate (P) uptake 1 *(Pup1)* gene confers P-deficiency stress tolerance in field-grown rice. A *Pup1* gene-specific SNP, indel-, and CAPS-based FMs have been developed to transfer this trait in P-deficiency-susceptible varieties through MAS [213]. Most areas of Asian countries are flooded during rice growing period in rainy seasons, and a tolerance gene Submergence-A1 *(SubA1)* for flooding has been identified. An FM for the *SubA1* gene has been developed and successfully used to transfer flooding tolerance in the “Swarna” variety of rice [212].

In foxtail millet, an SNP-based FM has been developed for the *SiDREB2* gene, which is responsible for a dehydration response [218].

Similarly, an FM for the *SbMATE* gene, which confers aluminum stress tolerance in sorghum, has been developed [216]. The *SbMATE* FM helps in the screening of sorghum germplasm for aluminum stress tolerance, which can be used in breeding. 

In cowpea, an FM for *CPRD12* gene conferring drought and salt tolerance has been developed [217].

## 7. Future Prospects and Conclusions

FM development for row crops has been implemented in plant breeding, but there has been meager FM research in horticultural and forage crops. Since FMs are derived chiefly from coding DNA within genes, these markers hold prospects for directly selecting a phenotypic trait. FMs also may be feasible for interspecies transferability and can be used in those species for which limited genomic resources are available. For successful implementation of precision breeding, the integration of advanced genomic tools with conventional plant breeding methods is essential. Moreover, development of cost-effective FMs is important for the efficient execution of precision breeding in crops. FMs can be effectively used in screening of germplasm, diversity analysis, QTL mapping, gene identification and isolation, and phylogenetic studies. In these studies, the use of FMs increases the accuracy and the efficiency of plant breeding for cultivar development with desired traits. In the future, allele-specific development of FMs for newly identified genes is essential for enabling efficient direct selection. Moreover, modern genomic and breeding approaches such as GWAS and GS have not been fully exploited for crop improvement but can be increasingly deployed in both model and non-model crop species with the availability of these NGS-based markers. In spite of available genomic resources, more work is needed to identify and develop FMs and to implement them in MAS for food security and sustainability goals. FMs may be used in the future for new plant breeding techniques using biotechnology in precision breeding.

In the future, we expect that new plant breeding techniques (NPBTs) using gene editing, cisgenesis, and epigenetic approaches will play an increasingly greater role in variety development. They can potentially confer traits that may be difficult via traditional breeding. Some examples include stress tolerance, shelf life, color, yield, and nutritional content. The results of conventional plant breeding are sometimes difficult to predict and require several years to fix traits in varieties. NPBTs may allow the breeders to develop improved varieties more precisely and more quickly. We expect that FMs can also come into play as NPBTs hit their stride to allow for efficient introgression of novel traits.

## Figures and Tables

**Table 1:** Candidate genes for functional markers (FMs) development for different traits.

Trait	Gene (s)	Chromosomal Location	Sequence	Crop	References
**Agronomic traits**					
Semi-dwarf stature	*Rht-B1 and Rht-D1*	4B, 4D	F-TCTCCTCCCTCCCCACCCCAAC R-CCATGGCCATCTCGAGCTGC & F-CGCGCAATTATTGGCCAGAGATAG R-CCCCATGGCCATCTCGAGCTGCTA	Wheat	[129]
Grain weight	*TaSus2-2B*	2	F-CGCCCTGAGCCG CATCCACA R-CGCTCGCCCGC CATTTATTTCTCT	Wheat	[118]
Grain weight	*TaGW2*	6	F-ATGGGGAACAGAATAGGAGGGAGGA R-CGAGTATGCCTAGAATGGAAAGAC	Wheat	[130]
Photoperiod response	*Phd-H1*	2	F-ACGCCTCCCACTACACTG R-CACTGGTGGTAGCTGAGATT	Wheat	[131]
Vernalization	*Vrn-D4*	5	F-CATAATGCCAAGCCGGTGAGTAC R-ATGTCTGCCAATTAGCTAGC	Wheat	[132]
Semi-dwarf	*sd1*	1	F-CACGCACGGGTTCTTCCAGGTG R-AGGAGAATAGGAGATGGTTTACC	Rice	[123]
Wide-compatibility gene	*S_5_^n^*	6	F-CGTCTTGCTTCTTCATTCCC R-GTAGGTAAACACAGGCAGAG	Rice	[133]
Photoperiod-thermo-sensitive genic male (PGMS and TGMS) sterility	*pms3 (p/tms12-1)*	12	F-GAATGCCATCTAAACACT R-ATTTTACTCTTGATGGATGGTC	Rice	[126]
Plant stature	*tb1*	1	F-CACATGAGCCCATGCCTCTC R-AAAGCGGTAAGTCCATGGGG	Maize	[134]
Plant height	*Dwarf8*	1	F-ACACTATCACCGCTCTATTG R-ACTCTTTCCCTGACTTCATT	Maize	[31]
Photoperiod response	*Phd-H1*	7	F-CCTCTTCGCTATTAC GCCAG R –GCCCTTCCCAACAGTTGCG	Barley	[135]
Vernalization requirements	*VRN-H 1*	5	F-TTCATCATGGATCGCCAGTA R-AAAGCTCCTGCCAACTACGA	Barley	[127]
Vernalization requirements	*VFR2*	A8	F-CTCGTAGCCCCGAGAACATC R-ACTCAAGCAACTTACCAAGTGGA	Brassica	[136]
Leaf hair number	*BrpHL1*	A9	F-TACTCCTCGTTCCCTCTGGG R-GGGGGAAATGCAGATTCCGA	Brassica	[128]
Seed development	*PvMIPSs and PvMIPSv*	1	F-TTGCCACGCACCTGCTAATA R-CCTGCAGCTGCGATTTTCAA	Common bean	[137]
Controlling flowering time	*MADS-box, Constants and Flowering locus T/Terminal Flower1*	16	F-ATGCACCTAGCCCAAGTGAC R-TGTTTGCATTCATGGCGTGT & R-ATCTGTTGTGCCGGGAATGT F-AACCGAAATGCAAAACAGGTGA	Pea, soybean, and burclover	[138]
Nodulation formation	*Rj2 and Rfg1*	16, 3	F-AAGTCTTAAATTGTGTTTGGATGGA R-TGAGAATTGTCACCACCGGG & F-AAGTCTTAAATTGTGTTTGGATGGA R-TGAGAATTGTCACCACCGGG	Soybean	[139]
Fruit size	*w2.2*	2	F-TCTGCTCAGAAGCATGCACA R-TTGTGACCTGTACCCCAGGA	Tomato	[140]
Short lateral branching	*slb*	11	F-CTTGCGCTCCTTGGTATTCC R-CAAGATCGGCAAGAGACAGC	Melon	[141]
Sutures on the rind	*s-2*	9	F-GCATCGGAATCTTGTTCGGC R-TCCGGTGGGAGATACCCAAT	Melon	[142]
Male sterility	*ms3*	5	F-GGTACTTTGA CCCTCATAATTGG R-TTGTTTGT GGTGTACG TGCT	Capsicum	[143]
Alternative respiration	*DcAOX1*	1	F-AAAATAACAATGATGATGACACG R-CTCCACTTCAGTGATATCCAA	Carrot	[144]
Curd architecture	*qCS.C6–1 and qCS.C6–2*	6C	F-CGGTACTGGAATGTGGACGT R-TGAATTGGTATGAACACGCCTC	Cauliflower	[145]
Early and late flowering	*BoFLC1.C9*	Unmapped	F-GGAAAGCAACATGGTGATGA R-CATGGTGTGAACCAGAGTCC	Cabbage	[146]
Male sterility	*CDMs399-3*	7C	F-TCCCTTTCACATCGTCCACA R-TGCAGCCCAGAACAGTGATA	Cabbage	[147]
Sex identification	*MYB35*	5	F-TTGCTTGGCGGATCATATTATG R-TTGCTTGGCGATGTCCCTTTTG	Asparagus	[148]
**Quality traits**					
Low molecular weight glutenin	*Glu-D3 and Glu-B3*	1D	F-CAGCTAAACCCATGCAAGC R-CAATGGAAGTCATCACCTCAA	Wheat	[149]
Yellow pigment content	*Psy1*	7A	F-ACATGCCGCTACTCCTATCC R-GTAGAGTGGCCAGACAAGGT	Wheat	[150]
Low molecular weight glutenin	*Glu-B3*	1B	F-ACAACAGGTTCAGGGTTCCA R-GCTATTTGGTGTGGCTGCAA	Wheat	[151]
Yellow pigment content	*TaZds-D1*	2D	F-ACATAGTCCTGACCGCCAAA R-AGAGTTGCTCCTTCCATGCT	Wheat	[152]
Lipoxygenase gene	*Talox-B1*	4B	F-ATGATACTGGGCGGGCTCGT R-TCAGATGGAGATGCTGTTGGG	Wheat	[153]
Fragrance	*badh2*	8	F-AGTTATGGTCTGGCTGGTGC R-TTGTGTGCTACCCACCCTTC	Rice	[154]
Fragrance	*nksbad2*	4	F-ATGGCAACATGGAAGGTAGC R-CATCAGCAAGCTCCAAACAA	Rice	[155]
Fragrance	*BADEX7-5*	8	F-TTAGGTTCTGAAGCCGGTGC R-TCCCAGTAAATGCAACCTAACAGA	Rice	[156]
Low glutenin content	*Lgc1*	10	F-TTCTACAATGAAGGCGATGC R-CTGGGCTTTAACGGGACT & F-ACCGTGTTATGGCAGTTT R-ATTCAAGGGCTATCGTCT	Rice	[157]
Fe and Zn	*OsNAS3, OsNRAMP1*	7	F-TCCATCGCTTGCTACCTCAC R-CCCGGAGATCGATCGAGACA & F-AGCACTCCCCCATCAATCAA R-ACTACACGGGTGGCTCTTTG	Rice	[158]
Intermediate amylose content	*Wx-in*	6	F-CAGCGTCGACGTAAGCCTAT R-CAGGCCCCTGAAATCCATGT	Rice	[159]
Oil content	*DGAT1-2*	6	F-TGGCTCTGCAATCAGGAGAA R-TGAAGCAGCAAACAACGAGC	Maize	[160]
Forage quality for digestibility	*Bm3*	4	F-TTCAACAAGGCGTACGGGAT R-AGTGGTTCTTCATGCCCTCG	Maize	[96]
Provitamin A	*ZmcrtRB3*	2	F-GTCGGTACTGGCAAGTGGAA R-TAGTACGTGGCCATGACGTG	Maize	[161]
Sweetness	*sugary1*	4	F-TCCCGACTTCAGAACGGTTG R-ACAACAGAGCAACCCCAACA	Maize	[162]
Provitamin A	*crtRB1 and LcyE*	10,8	F-CACAGGTCGCTGCGTACTTA R-GGGAGACAGCTCACAGGAAC & F-CAGTGCGCTGAAGGCTACTA R-GGATGAAAGGGTCGAGCCAA	Maize	[163]
Soluble acid invertase	*SAI-1*	4	F-GGATTCCACTTCCAGCCACA R-CGACGGGGTAGAAGTCGATG	Sorghum	[164]
Fragrance	*SbBADH2*	4	F-CGCAGTAGTGGAGTGGTTGT R-ACTGTGGCGGTTCTTGCATA	Sorghum	[165]
Fragrance allele	*Gmbadh2-1 and Gmbadh2-2*	5	F-GTGATCTGCGAGGGAGGGAG R-TGAGTTGCAGGCAGTGTCAT	Soybean	[166]
White flesh	*wf*	9	F-TTGGAGGTTCAATGCTTGCC R-CAAAGACCAGAGCACCATCG	Melon	[167]
Green flesh color	*gf*	8	F-TCTGCAAAATGGTTGCTTTGAA R-AGGTGGATGTGGCACACAAA	Melon	[141]
Flavonoids	*AgFNSI*	4	F-ATGGCTCCATCAAC TATAAC R-CTGCCCTGGCAATCTCCG	Celery	[168]
Starch content	*NnHXK and NnGBSS*	Unmapped	F-TCTAAATCCCAATCCGTCC R-GCACGAACTCTTGGCAATC	Lotus	[169]
Pungency	*Pup1*	2	F-CCATGGATTGTTGCTCGGGCCTCC R-CCGTACCGCCCCATTGCGATTCC	Chilli	[170]
Anthocyanin biosynthesis	*VfTTG1*	Unmapped	F-TATGAATTCATTTTTAGTTCCCACCTAAC R-GTATCCGGTTGAGGACTCTCATAGATA	Faba bean	[171]
β-Carotene & Flesh	*QA/QC*	3	F-AGTGCGGGACAAGATGATCA R-TCCCGAACATCTGAGCAAGT	Sweet potato	[172]
Carotenoids	*b_CHYβ-1*	Unmapped	F-TCCAGCTTGGGAATTACGTC R-ACAACGAAGCGTGCCATAG	Sweet potato	[173]
**Biotic stresses**					
Powdery mildew	*Pm3*	1A	F-CAAGTACCAACCACAGCCAC R-CCATTGCAACCACAGGAACA	Wheat	[174]
Stem rust resistance	*Sr45*	1D	F-GTCCATTTTACGACGGTCCG R-CTGGTCGGTAGGGAAGCTAG	Wheat	[175]
Bacterial blight resistance	*Xa3*	11	F-GAATGGGTGGGGTTGGGAAG R-CCATGCACGCTTGTCGAATC	Rice	[176]
Bacterial blight resistance	*xa5*	5	F-ACGGAGTTGCAATGTTGCTG R-GGCCAGGAGTAAAGCGGATT	Rice	[177]
Bacterial blight resistance	*xa13*	8	F-GGCCATGGCTCAGTGTTTAT R-GAGCTCCAGATCTCCAAATG	Rice	[178]
Bacterial blight resistance	*Xa21*	11	F-AGACGCGGAAGGGTGGTTCCCGG R-AGACCGGTAATCGAAAGATGAAA	Rice	[179]
Bacterial blight resistance	*Xa38*	4	F-TCTTCTATTGCTAACATTGGTG R-AGCGTAAGTAAAAGTCTC	Rice	[180]
Brown plant hopper resistance	*Bph14*	3	F-CAATCCGAGCTTACGTGGTG R-GGTGGAGAAGGCAAGAGTCT	Rice	[181]
Blast resistance	*Pit*	1	F-GTGACGGAAGTGCATGGGTA R-ACCAGGGAACCCGACAAGAA	Rice	[182]
Blast resistance	*Pi54 (Pik^h^)*	11	F-CCTCTTGAGTTGAATTGGCACG R-CCTCGTGCAGCTGTTTTCAC	Rice	[183]
Blast resistance	*Pi35*	1	F-TCCATGGCGGAGGTGGTGTTGGCTG R-AGAGCAAATCTTGGGGTGTCTGCAA	Rice	[184]
Blast Resistance	*PigmR*	6	F-ATGTCGGAGGAAGCAGGTC R-ATGTCACGCAGCAAAACCAT	Rice	[185]
Brown plant hopper resistance	*Bph9*	12	F-CACTCGCACGGATACAATGG R-GATCGTGACACATGCATGCT	Rice	[186]
Powdery mildew	*NBS–LRR class of resistance genes*	2	F-CGTTTTGTATGGCGTCCGAT R-TTGTCGCTGAGGTCCATCTT	Barley	[187]
General stress response	*ERF transcription factors*	1	F-ACAGTGGTGGCAAGTGTGAA R-ACGGCCTCCTTCTTACTCCT	Several crops	[188]
Leaf rust resistance	*Rph7*	3H	F-TGGAAACCACTGTACAGCCT R-CAGGCATGGGAGTGAACCTA	Barley	[189]
Tomato yellow leaf curl virus	*ACY*	6	F-CCTTATGATGTCTCGTGAAAGG R-GAAGCACAGATTGAAGAAAACC	Tomato	[190]
Bacterial wilt	*Bwr-6, Bwr-12*	6,12	F-TCAAGGTCCACTACCTTCATCC R-TCGGTATAGAGGGTACGTTG	Tomato	[191]
Fusarium wilt	*frl*	9	F-TACGATGACGTCGGT R-ATGCTACTGCGATGAAAC	Tomato	[192]
Fusarium wilt	*Fom 1*	7	F-AACGAGAAGGCGGTGGAAAT R-CGATCTCCTCAAGGGAAGGTG	Melon	[193]
Leaf scald resistance	*Rpf*	Unmapped	F-TTGTTGGAACCTTTCGCTGG R-TAGACCTGTGCTGCCGTAAA	Sugarcane	[194]
Powdery mildew resistance	*Pm-2 F*	1	F-GCCCAACCTTCAACTCGATA R-TTGAATCTCATTTTTCTGTTGCAT	Melon	[195]
Melon necrotic spot virus	*nsv*	4	F-GTTTCTGATACGATGTTGTTTCCCTG R-GCCGAGATGCAGCAGGATGCTTTGCAC	Melon	[196]
Zucchini yellow mosaic virus (ZYMV)	*eIF4E*	3	F-TGGACITTYTGGTTYGAYAA R-GGRTCYTCCCAYTTIGGYTC	Watermelon	[197]
Powdery mildew	*Pm*	5	F-ATTTTCTTGCTTCAAATGGA R-ATAAGCAAAAGCATCGAAAG	Watermelon	[198]
Powdery mildew	*Pm-s*	5	F-CCCTATGCGTGAAAGCCACT R-CGCCTCAAACCCATACCCAA	Cucumber	[199]
Cauliflower mosaic virus (CMV)	*cmv6.1*	6	F-ACAAAGCTTCTCCGCAAATG R-GGAGGGAAAGGAAGGAGAGA	Cucumber	[200]
Bacterial wilt-resistance	*S401*	6	F--G ACTGCGTACCAATTCAGTT R-GATGAGTCCTGAGTAACACGATG	Eggplant	[201]
Powdery mildew resistance	*InDel 1*	1	F-AACTTGGTAGCAATTTTATTGGGT R-TGGAGACAATGTGCATAAGTCTCT	Capsicum	[202]
Bacterial leaf spot resistance	*Xcvr*	2	F-TATCAAACGTAAAGTTGGAGCTTGT R-CCAAACACCTTGTGCATTGCT	Lettuce	[203]
Turnip mosaic virus resistance	*retr02*	4A	F-GGAGAAGACAAACAAACCCCC R-A TACCTTCGACACCGTCCAAGACTT	Turnip	[204]
Powdery mildew resistance	*er1-7*	6	F-CGACACCGTATTCAAGCAGG R-TGTTGCCCTGTTTGATCGTT	Pea	[205]
Mungbean Yellow Mosaic Virus	*YR4*	Unmapped	F-ACAAACATGGGCTGGAACAC R-GTGCCTGTAACTGCTCACAC	Mung bean	[206]
Resistance to weevils	*VrPDF1*	Unmapped	F-CCAAGCTTGGTTAACAGTTTCTAGTGCACC R-GCGTCGACGATGGAGAAGAAATCACTGGCC	Mung bean	[207]
**Abiotic stresses**					
Dehydration tolerance	*TaMYB2*	Unmapped	F-GAGGCCAGCTAGCAGCTGCC R-ATTGCCGGACGCGCAAGAGG	Wheat	[208]
Drought stress tolerance	*TaAQP*	Unmapped	F-ACATCAATTTTACCGTGCTTTG R-CAATCAATCTGCCGACTGTG	Wheat	[209]
Drought stress tolerance	*DREB1*	3D	F-GAATGGATCCCGGAAAGCAC R-GGGAATGAACCAAGCCACAG	Wheat	[210]
Salt tolerance	*Tt* *ASR1*	Unmapped	F-ACCCCTACTTCTACATGCCG R-ATGATGGAGCTGTGGGACG	Wheat	[211]
Submergence tolerance	*SubA1*	9	F-CTAGTTGGGCATACGATGGC R-ACGCTTATATGTTACGTCAAC	Rice	[212]
Tolerance to phosphorus (P) deficiency	*Pup 1*	12	F-CTGGACTTGACCCCAATGTA R-TCTGATGGAGTGTTCGGAGT	Rice	[213]
Drought stress tolerance	*OsSAPK2*	5	F-AAGGACATAGGGTCGGGGAA R-TGGCCAAATGTGTGGGAGTT	Rice	[214]
Drought tolerance	*MYBE1*	5	F-GGTACCCTGTCAAGGTTCGG R-AATTACTGGCCCCAGGTTCG	Maize	[215]
Photoperiod response	*Phd-H1*	2H	F-GTTGAGATCGACAGTCCCCA R-GGGCTCCTATCTCCAACTCC	Barley	[135]
Aluminum stress tolerance	*SbMATE*	4	F-TAAGGCGCAATCATCATGGC R-CAACAAGATTCTGGAGCCGG	Sorghum	[216]
Drought and salt stress tolerance	*CPRD12*	11	F-AAAGCATGCCCTAGTGGGAC R-ATGTCGGAAGCTACGGTTTCT	Cowpea	[217]
Dehydration response	*SiDREB2*	Unmapped	F-CAACGGACTTGGGGCAAATG R-ATCGTTCGCTTCTGCCTTCA	Foxtail	[218]
Salinity tolerance	*Salt index_QTL 1*	Unmapped	F-TGTACACTGTGTTTCTGTTGGT R-GTATTCGATCGTCCCTCCCG	Field pea	[219]
